# Obituary Prof. Dr. med. habil Peter Karl Lommatzsch (20.12.1934–23.11.2020)

**DOI:** 10.1007/s00417-020-05069-w

**Published:** 2021-09-27

**Authors:** Rudolf F. Guthoff, Antonia M. Joussen, Norbert Bornfeld

**Affiliations:** 1 Department of Ophthalmology, University of Rostock, Rostock, Germany; 2 Department of Ophthalmology, Charité - Universitätsmedizin Berlin, Berlin, Germany; 3 Department of Ophthalmology, University of Duisburg-Essen, Essen, Germany

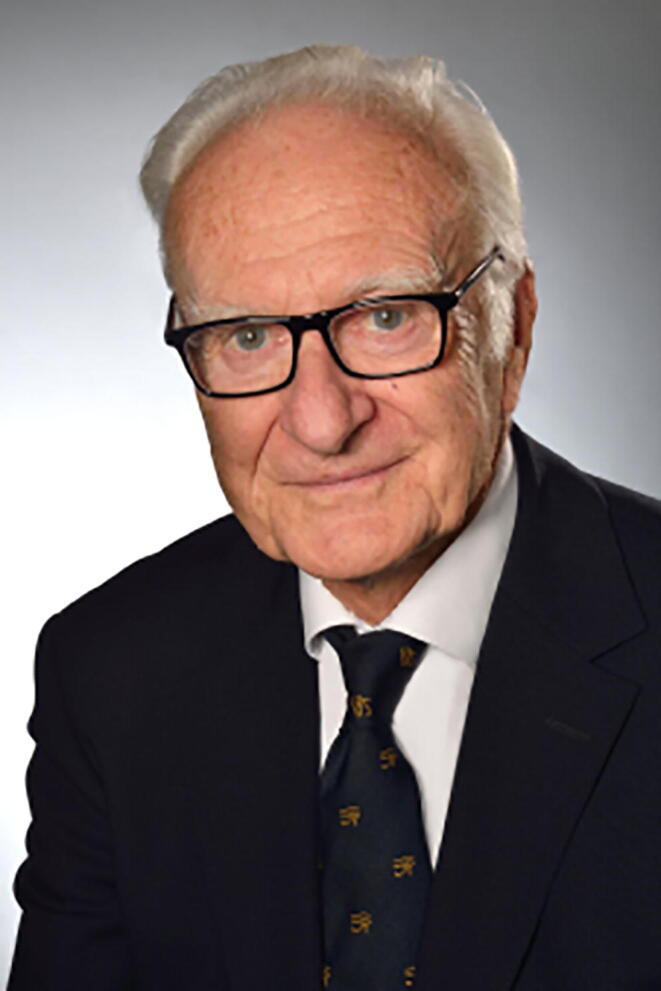



Prof. Peter Karl Lommatzsch died in Leipzig on 23.11.2020 at the age of 85. He was born on 20 December 1934 in Saxony, studied medicine from 1952 to 1957 at the Karl-Marx-University Leipzig together with his lifelong friend Gottfried Naumann, obtained his doctorate in 1957, and went to the Eye Clinic of the Humboldt University Berlin (Charité) as a research assistant. He finished his Ph.D. thesis on “*The Use of Beta-applicators for the Treatment of Intraocular Tumours*” in 1963 during the chairmanship of Prof. Karl Velhagen. From 1976 to 1982, he was Professor and Chair of the Eye Clinic of the Municipal Hospital in Berlin-Buch. In 1980, he was a visiting Professor at the University of Iowa and in 1981, he was appointed full Professor at the Karl-Marx-University in Leipzig and Director of the University Eye Clinic. In 1992, he left university service and worked as an ophthalmologist in independent practice in Leipzig until 2005. During this time, he was also engaged as an eye surgeon in Ngaoundere (Cameroon).

Professor Peter Lommatzsch was one of the most innovative and internationally renowned ophthalmologists of his generation. The ruthenium applicators he developed together with G. Vormum at the former Academy of Sciences of the former GDR (German Democratic Republic) were one of the most important further developments in eye-preserving radiotherapy of intraocular tumours. Peter Lommatzsch pioneered work in basic scientific research on the radiosensitivity of the eye and its adnexae, which still defines the standard for further development of radiotherapy for ocular tumours today. The scientific work of more than 130 original papers over a period of 40 years covers the entire range of intra- and extraocular tumours and has enabled ground-breaking progress in the treatment of malignant melanomas of the uvea and conjunctiva, retinoblastomas, and intraocular vascular tumours. In addition to this scientific focus, Peter Lommatzsch was one of the first to introduce modern iris and capsular bag–supportedartificial lens surgery in the former GDR through his contacts with Barraquer (Barcelona), Fjodorov (Moscow), and Binkhorst and Worst (Netherlands), and was intensively involved in establishing modern vitreoretinal surgery in the former GDR.

Whoever worked with Peter Lommatzsch found in him an overwhelmingly stimulating conversational partner and motivator, with an open mind for all questions of ophthalmology and beyond. Under his leadership, the Centre for Ophthalmic Oncology and Microsurgery was established in Berlin-Buch and later in Leipzig. Colleagues from Eastern and Western countries adopted ruthenium-beta radiation therapy for the benefit of patients in their home countries. Despite the difficult political environment, Peter Lommatzsch maintained intensive scientific and personal contacts with university hospitals in Hamburg, Essen, and Munich, and was always available with advice and experience. His high international reputation became known during his guest professorships in Iowa City with Prof. Blodi and in Leiden with Prof. Osterhuis as well as being an elected member in the Club Jules Gonin.

In the middle of the Cold War period, he succeeded in bringing together almost all important ocular oncologists from the East and West for the first “International Symposium on Intraocular Tumours” in Schwerin in 1981. This congress was the first in a continuing series of congresses that led to the founding of the “International Society of Ocular Oncology”. Furthermore, Peter Lommatzsch was co-founder and president of the European Ophthalmic Oncology Group. Until the end of his life, Peter Lommatzsch remained scientifically connected to ocular oncology and actively participated in the latest developments in the field of eye salvaging therapy and molecular genetics.

Peter Lommatzsch was awarded the Graefe Prize of the German Ophthalmological Society (2002) for his scientific excellence and was elected into the “Hall of Fame Ophthalmology” of the German Society of Ophthalmic Surgeons (2015).

Peter Lommatzsch has not only been involved in ophthalmology. Another main focus of his commitment was the protection of birds and the preservation of rare species, for which he worked with great physical and social commitment until his death.

With Peter Lommatzsch, we have lost one of the great innovators and stimulators of European ophthalmology, who never lost the joy of his subject of ophthalmology, even in politically difficult times, and who was also highly regarded as a mentor and scientist after his university appointment in Leipzig. In 2008, he writes in retrospect

“On 1 April 2005 I ended my ophthalmological activity. I do not look back in anger, but am happy every day that my children and grandchildren have the great fortune to be able to live in freedom. Anyone who, like me, has lived for more than half a century only under totalitarian regimes does not long for the past times of bondage, even under the new challenges of our time such as globalisation, brutalisation through the excesses of predatory capitalism and the closely related threatening clash of civilisations. If my fate in Leipzig in 1992 was the price of living in freedom, I was happy to pay it for my children”.

Peter Lommatzsch concludes his autobiographical essay with a quotation from the Leipzig anatomist Kurt Hermann Alverdes, whom he held in high esteem, who stated in his anatomy lecture on the occasion of the unrest and political reprisals of 1953: “Remember, ladies and gentlemen, the mentality of the people remains almost always the same under all political systems”.

